# The paradoxical relationship between ligamentum flavum hypertrophy and developmental lumbar spinal stenosis

**DOI:** 10.1186/s13013-016-0088-5

**Published:** 2016-09-05

**Authors:** Prudence Wing Hang Cheung, Vivian Tam, Victor Yu Leong Leung, Dino Samartzis, Kenneth Man-Chee Cheung, Keith Dip-Kei Luk, Jason Pui Yin Cheung

**Affiliations:** 1 Department of Orthopaedics and Traumatology, The University of Hong Kong, Hong Kong, SAR China; 2 School of Biological Sciences, The University of Hong Kong, Hong Kong, SAR China

**Keywords:** Developmental spinal stenosis, Ligamentum flavum, Hypertrophy, Fibrosis

## Abstract

**Background:**

Ligamentum flavum (LF) hypertrophy is a common cause of lumbar spinal stenosis and is thought to be degeneration-driven. Developmental spinal stenosis (DSS) is characterized by pre-existing narrowed spinal canals and is likely a developmental problem that occurs in childhood. In these cases, the LF may demonstrate different characteristics as compared to degeneration-driven stenosis. Thus, this study aimed to investigate the relationship between histological changes of LF and canal size.

**Methods:**

Patients who had surgical decompression for lumbar spinal stenosis were prospectively recruited and divided into three groups (critical DSS, relative DSS and non-DSS) based on previously defined anteroposterior bony spinal canal diameter measurements on MRI. The degree of disc degeneration and LF thickness were also measured from L1 to S1. Surgical LF specimens were retrieved for histological assessment of fibrotic grade and area of fibrosis.

**Results:**

A total of 19 females and 15 males (110 LF specimens) with an overall mean age of 65.9 years (SD ± 9.8 years) were recruited. DSS was found to have a significant negative correlation (*p* < 0.001) with LF thickness, its fibrotic grade and area of fibrosis (%). Non-DSS exhibited a significant positive relationship with the degree of LF fibrosis. Disc degeneration and LF thickness had no correlation with LF histology.

**Conclusions:**

Our study is the first to definitively note that degeneration is the cause of LF fibrosis in non-DSS patients; however, in contrast, an inverse relationship exists between canal size and LF fibrosis in DSS patients, suggesting a different pathomechanism. Hence, despite a similar degree of LF thickness, DSS patients have LF with less fibrosis compared with non-DSS patients. Further investigation of the cause of LF changes in DSS is necessary to understand this relationship.

## Background

Patients with lumbar spinal stenosis present with neurogenic claudication, radiculopathy and/or neurological deficit due to compression of the neural tissue in the spinal canal [1]. For patients who are unresponsive to conservative measures, such as anti-inflammatory medications, physiotherapy and epidural steroid injections, decompression surgery should be offered as the primary treatment modality, especially for those with severe symptoms [2]. Unfortunately, there is an increased risk of reoperation due to recurrence of stenotic symptoms at the surgical site or at adjacent levels [3]. Revision surgeries are undesirable as patients experience less favourable outcomes than in the index operation [4]. Therefore, in order to minimize the occurrence of suboptimal surgical outcomes, it may be beneficial preoperatively to identify patients who are at higher risk of reoperation. Such at-risk groups are patients with developmental spinal stenosis (DSS), who may have poorer prognosis with risk of reoperation.

Developmental spinal stenosis is characterized as pre-existing narrowed bony spinal canals originating from the mal-development of the dorsal spinal elements [5, 6], and developmental factors have been emphasized as the primary cause of spinal stenosis [7]. This results in reduction of space available for accommodating neural contents, increasing not only the risk of developing stenotic symptoms, but also symptom recurrence requiring repeated surgery at other involved levels [8]. Previous studies comparing symptomatic patients who underwent surgery and asymptomatic individuals have noted that the anteroposterior (AP) bony spinal canal diameter is the most relevant magnetic resonance imaging (MRI) measurement associated with DSS [9]. A MRI-based study has also demonstrated that these lumbar spinal canal dimensions can be assessed on both T1- and T2- weighted MR scans [9]. Hence, based on MRI, level-specific cut-off values of each vertebral level have been identified to assist with the diagnosis of DSS [10].

One major contributor of neural compression in lumbar spinal stenosis is ligamentum flavum (LF) hypertrophy [11]. With narrowing of the bony spinal canal, there may be a lower threshold for the severity of LF hypertrophy. Two theories exist for the appearance of LF hypertrophy. Firstly, LF hypertrophy may be due to a fibrotic change [12, 13], which is a result of the degenerative cascade and is characterized by increased level of collagen fibers with reduced amount of elastin or degenerated elastin [14]. In lumbar spinal stenosis, LF has been reported to demonstrate both accelerated collagen synthesis as well as elastic fiber degradation [15]. An increased number of fibrocartilaginous cells are found, resulting in the proliferation of collagen fibers within a hypertrophic LF [16]. This is in comparison to normal LF, which consists of 80 % elastin and 20 % collagen [17]. In this case, LF hypertrophy alone compresses neural elements, even without annulus fibrosus bulging or nucleus pulposus herniation [1]. The second pathomechanism is the apparent LF hypertrophy as a result of disc height reduction or disc degeneration [18]. A loss of disc height will cause laxity in the ligamentous tissues surrounding the spinal column [19], which leads to LF buckling.

It is therefore clear that both spinal canal diameter and LF thickness play crucial roles in the pathogenesis of spinal stenosis. However, the relationship between canal size and LF thickness is unknown. Due to a pre-existing narrowed canal, the neural elements are more susceptible to even milder degrees of LF hypertrophy. By comparison, in larger-sized canals, a more significant contribution may exist with LF hypertrophy. Thus, this study aims to investigate the relationship of LF thickness with spinal canal diameter, and whether the LF hypertrophy can be accounted for by the development of fibrotic changes or secondary to reduced intervertebral disc height.

## Methods

This was a prospective study of patients with symptomatic lumbar spinal stenosis who were treated with decompression surgery at a single institution from June 2014 to April 2015. Ethics approval was obtained from the local institutional review board. All patients had failed conservative treatment, including at least three months of physiotherapy and analgesics, prior to surgery. Patients with isthmic and degenerative spondylolisthesis, scoliosis, fractures, previous surgery and epidural injections which might affect the quality of the LF specimens, infections and tumors were excluded. Patient particulars including age, gender, body mass index (BMI), degree of disc degeneration and radiographic measurement were collected for analysis.

### Spinal canal diameter and LF thickness

**Fig. 1:**
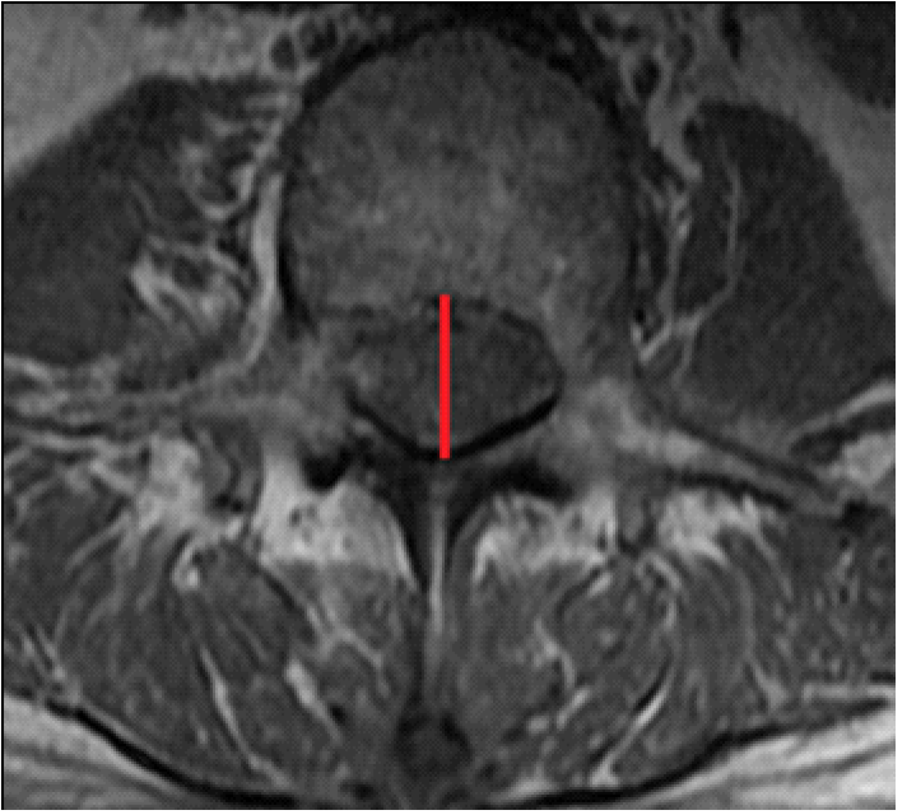
Measurement of midline anteroposterior (AP) spinal canal diameter at L1 to S1 using T1-weighted axial MRI scan (marked in red line) - at the cut where the entire bony canal ring could be seen and with the thickest pedicle width

**Fig. 2:**
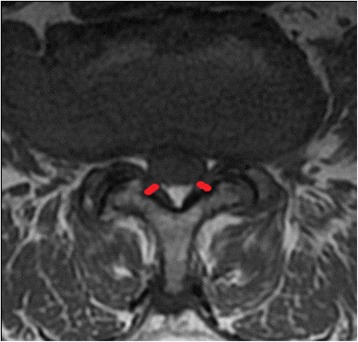
Measurement of ligamentum flavum (LF) thickness using T2-weighted axial MRI scan (marked in red lines) - at the cut with the thickest LF measured at the midpoint of the facet joint level

Measurements of spinal canal diameter and LF thickness were extracted from preoperative T1- and T2-weighted axial MRI scans. A 3T MRI was obtained of all subjects. The field of view was 18 x 18 cm for axial scans and 28 x 28 cm for sagittal scans. Slice thickness was 4 mm for both scans, and slice spacing was 0 mm for axial scans and 0.5 mm for sagittal scans. Imaging matrix was 288 x 192 for axial scans and 512 x 224 for sagittal scans. The repetition time was 700 to 800 ms and 4000 to 6000 ms for T1 and T2, respectively. The echo time was 8 to 10 ms and 80 to 100 ms for T1 and T2, respectively. There were 11 slices per vertebral level and parallel slices were made according to the disc and pedicle levels. A spine surgeon blinded to the patient details performed all measurements using Centricity Enterprise Web V3.0 software (GE Medical Systems, 2006). All canal diameter measurements were performed first for all patients and then all LF were measured after the order of images measured were randomly selected. Spinal canal diameters at L1, L2, L3, L4, L5 and S1 were assessed at the vertebral level (Fig. 1). Due to its anatomical location, LF thickness was measured at L1-L2, L2-L3, L3-L4, L4-L5 and L5-S1 intervertebral disc levels. The AP spinal canal diameter (Fig. 1) was measured using T1-weighted axial MRI, at the cut where the entire bony canal ring could be seen and with the thickest pedicle width [9]. The LF was measured (Fig. 2) at the midpoint of the facet joint level using T2-weighted axial MRI at the cut with the thickest LF measured.

### Disc degeneration

As disc degeneration could be a possible cause for spinal canal narrowing and might be a confounder to the lumbar spinal stenosis assessment, it was used to stratify our results. Disc degeneration was characterized by a reduction in disc height. Thus, the disc height was assessed on T2-weighted MRI sagittal images to correlate with other imaging findings. Disc height was measured from the middle of the intervertebral disc (from the thickest cut) perpendicular to the endplate, inclusively from L1-2 to L5-S1 at each level. Any anterior disc bulging was also noted.

### Classifying patients

As this study aimed to investigate the characteristics of LF corresponding to canal size, it was necessary to differentiate patients based on the origin of neural compression and whether there was an existing narrowed canal. Based on the phenotypical definitions of DSS by Cheung et al., [10] patients were classified into 3 groups: DSS with critical stenosis (Group 1), DSS with relative stenosis (Group 2), and non-DSS (Group 3). Measurements were based on the AP bony spinal canal diameter at the vertebral level. Critical stenosis values were defined as: L4 < 14 mm, L5 < 14 mm, and S1 < 12 mm. DSS was defined as: L1 < 20 mm, L2 < 19 mm, L3 < 19 mm, L4 < 17 mm, L5 < 16 mm, and at S1 < 16 mm.

### Histological assessment

Excised LF was obtained during surgery at each operated level and was separated into left and right sides. Excised LF was fixed in 4 % (weight/volume) paraformaldehyde in phosphate-buffered saline (pH 7.4) immediately upon removal, then stored at 4 °C for 48 h before processing, and embedded in paraffin wax blocks longitudinally or in cross-section. Specimens were prepared in 5 μm sagittal sections according to the excised level and sides using a microtome.

**Fig. 3:**
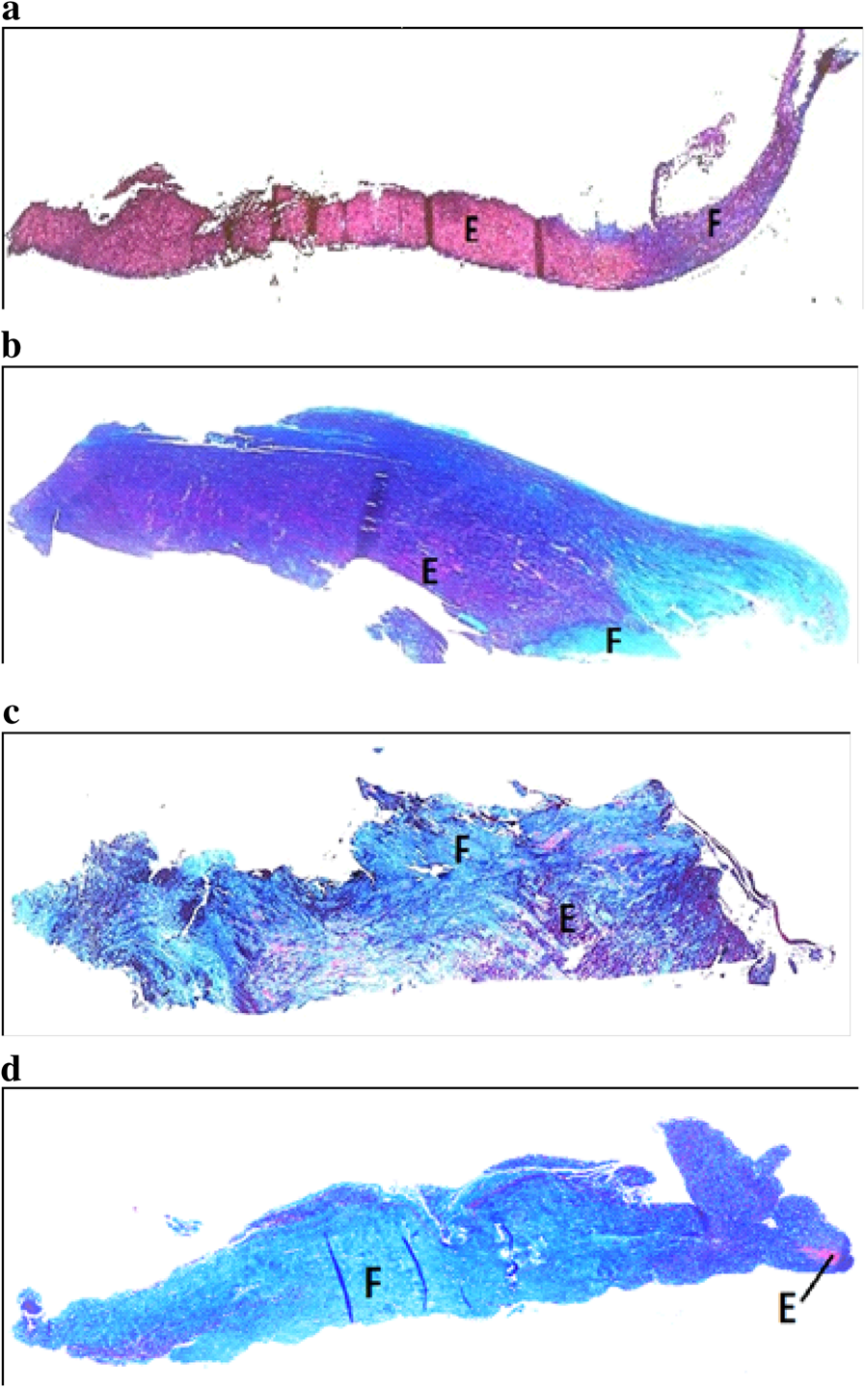
Histological assessment of fibrotic changes in ligamentum flavum (LF). Fibrotic Grade 1 (**a**), Fibrotic Grade 2 (**b**), Fibrotic Grade 3 (**c**), Fibrotic Grade 4 (**d**). LF sections were stained with Masson’s trichrome staining and imaged at 4X magnification under microscope. Areas marked with *"E"* signify keratin/elastin/muscle fibres (red stain), and *"F"* signifies collagen fibres (blue stain). There were no developmental spinal stenosis (DSS)/ non-DSS subjects with a fibrotic grade of 0 as all were stenotic patients

Masson’s trichrome staining was performed for each LF specimen using the protocol by Carson [20], with the main staining solutions being Weigert’s Haematoxylin, Biebrich Scarlet-Acid Fuchsin and Aniline Blue working solutions. Nuclei, collagen fibres and keratin/elastin/muscle fibres were represented by black, blue and red colors, respectively. The slides were viewed under Nikon Eclipse 80i microscope and microscopic images were captured by computer imaging software (NIS-Elements F4.30.01 64-bit, Nikon, Japan). Each LF specimen was assessed for the degree of fibrosis, which was represented by the area occupied by collagen fibers out of the entire cut section. This was calculated using ImageJ 1.48d (RSB, NIMH, Maryland, USA), and was expressed in percentages. For the ease of comparison, grading system (Grade 0–4) defined by Sairyo et al. [21] was also used to indicate histological findings: Grade 0 represents normal tissue with no fibrosis, Grade 1 represents fibrosis less than or equal to 25 % of the entire area, Grades 2 and 3 represent fibrosis occurring between 25–50 % and 50–75 % of the entire area, respectively, and Grade 4 represents fibrosis of more than or equal to 75 % of the entire area (Fig. 3).

### Statistical analysis

SPSS version 21.0 (Chicago, USA) was used for descriptive and frequency analyses. Mean and standard deviation (±SD) scores were performed where applicable. Correlation analyses of the thickness of LF (right and left sides), spinal canal diameter, disc height, BMI and degree of fibrosis were performed using Pearson’s correlation coefficient. In the correlation analyses, an “r” value closer to the value of “1” represented an excellent/perfect correlation. Paired samples t-test and one-way analysis of variance (ANOVA) were performed for comparison of correlations of LF thickness and fibrosis between study groups, as well as mean value comparison between the three groups. *P*-values of <0.05 were considered significant and 95 % confidence intervals were considered to assess the degree of precision.

## Results

A total of 34 patients (19 females, 15 males) were recruited for this study. Females had a mean age of 67.1 ± 10.4 years and males had a mean age of 64.4 ± 11.0 years. A total of 380 spinal canal diameters, along with 190 intervertebral disc heights and LF thickness were assessed from MRI scans. The most common involved level for surgery was L4-5 (34.5 %). Despite the significant differences found in spinal canal diameter (*p* < 0.001) among all three study groups, LF thickness, disc height and BMI did not demonstrate any differences. When comparing levels in common, the disc height and BMI had no significant differences (Table 1), whereas the difference in canal diameter was 10.2 mm at L4, and 4.8 mm at L5 (Table 2) for Groups 1 and 3. Anterior disc bulging occurred most commonly within Group 3 (71.4 % of all non-DSS) as compared to Group 2 (49.2 %) and Group 1 (47.8 %).

**Table 1:** Study group characteristics

	Group 1 (Critical DSS)	Group 2 (Relative DSS)	Group 3 (Non- DSS)	*p*-value
Vertebral Levels	27	148	15	
Gender	F: 11	F: 19	F: 4	0.65
	M: 6	M: 10	M:10	
Mean Age at Surgery (Years (±SD))	F: 68.0 (11.0)	F: 67.1 (10.4)	F: 64.5 (9.0)	0.26
	M: 67.0 (10.3)	M: 62.1 (12.9)	M: 47.4 (26.8)	
Mean LF thickness (mm (±SD))	R: 3.8 (1.4)	R: 3.4 (1.7)	R: 3.1 (0.6)	R: 0.45
	L: 4.0 (1.7)	L: 3.4 (1.6)	L: 3.3 (1.1)	L: 0.30
Mean Spinal Canal Diameter (mm (±SD))	12.7 (1.3)	15.4 (1.3)	17.7 (2.6)	<0.001*
Mean Disc Height (mm (±SD))	10.4 (2.3)	9.9 (2.5)	9.7 (2.9)	0.58
BMI (±SD)	22.8 (2.3)	24.3 (3.2)	26.7 (3.7)	0.06

*LF* ligamentum flavum, *BMI* body mass index, *SD* standard deviation, *F* female, *M* male, *R* right, *L* left

*significant difference between three study groups

**Table 2:** Imaging findings of study groups

Study Group	Group 1 (Critical DSS)		Group 2 (Relative DSS)					Group 3 (Non-DSS)	
Levels	L4	L5	L1	L2	L3	L4	L5	L4	L5
Mean Spinal Canal Diameter (mm (±SD))	13.1 (1.2)	12.9 (0.5)	16.5	14.2 (1.0)	15.2 (1.5)	15.5 (0.8)	15.2 (0.4)	23.3	17.7 (1.8)
Levels	L4-L5	L5-S1	L1-L2	L2-L3	L3-L4	L4-L5	L5-S1	L4-L5	L5-S1
Mean LF thickness (mm (±SD))	R: 4.4 (1.6)	R: 3.3 (1.8)	R: 6.1	R: 4.8 (1.8)	R: 4.0 (1.8)	R: 3.5 (1.6)	R: 4.5 (1.7)	R: 4.2	R: 3.0 (0.3)
	L: 4.6 (1.9)	L:3.4 (1.5)	L: 2.5	L: 4.3 (1.5)	L: 4.1 (1.8)	L: 4.3 (2.1)	L: 3.5 (1.2)	L: 5.3	L: 3.2 (0.4)
Mean Fibrosis Area (% (±SD))	R: 48.6 (25.5)	R: 38.5 (16.2)	R: 15.3	R: 33.9 (17.8)	R: 52.6 (20.6)	R: 47.4 (17.8)	R: 41.8 (19.2)	R: 72.6	R: 59.5 (22.2)
	L: 57.7 (18.2)	L: 57.9 (6.4)	L: 15.3	L: 70.0 (5.4)	L: 52.8 (24.9)	L: 43.5 (16.9)	L: 48.9 (29.4)	L: 66.5	L: 52.7 (27.1)

*LF* ligamentum flavum, *SD* standard deviation; %: percentage, *R* right, *L* left

### Correlation of LF thickness and spinal canal diameter

In Group 3, LF thickness was strongly correlated to the spinal canal diameter (Right: *r* = 0.98, p ≤ 0.001; Left: *r* = 0.92, p ≤ 0.01), and a weaker relationship for Group 2 at L5-S1 only (*r* = 0.75, *p* < 0.005). Group 1 failed to demonstrate any significant correlation of LF thickness and canal diameter at all, despite its pre-existing narrower canal space.

### Correlation of LF thickness and fibrosis

There were 55 operated intervertebral levels, giving rise to a total of 110 LF specimens collected. With comparable mean LF thickness according to individual intervertebral levels as expressed in Table 2, a significant *negative* correlation of LF thickness and fibrotic grades was found in both Groups 1 and 2 (Table 3).

**Table 3:** Correlation of surgical ligamentum flavum thickness and fibrosis

	Right LF *r*-value	*p*-value	95 % CI	Left LF *r*-value	*p*-value	95 % CI
(a) Correlation of Surgical LF Thickness and Fibrotic Grades						
Group 1	-0.29	0.028	0.24–3.26	-0.42	0.069	-0.14–3.06
Group 2	-0.32	<0.001*	1.04–2.44	-0.22	<0.001*	0.84–2.21
Group 3	0.49	0.18	-0.42–1.46	0.72	0.059	-0.88–2.49
(b) Correlation of Surgical LF Thickness and Area of Fibrosis (%)						
Group 1	-0.42	<0.001*	-59.11–-23.73	-0.31	<0.001*	-65.37–-41.31
Group 2	-0.41	<0.001*	-47.88–-34.39	-0.29	<0.001*	-53.87–-38.27
Group 3	0.65	0.008	-89.55–-29.46	0.50	0.019	-88.48–-16.41

LF: ligamentum flavum, CI: confidence onterval, %: percentage, r: correlation coefficient, * significant *p*-value; Group 1: critical developmental spinal stenosis (DSS), Group 2: relative DSS, Group 3: Non-DSS

The LF thickness was also found to have an *inverse* relationship with the area of fibrosis (%) in Group 1 (Table 3). On the contrary, Group 3 had a significant *positive* correlation of LF thickness and fibrosis area. These relationships were even stronger when analyzing particular intervertebral levels, especially at L4-5 for Groups 1 and 2 demonstrating significant negative correlations, and at L5-S1 where Group 2 demonstrated an inverse relationship and Group 3 exhibited a very strong positive relationship (Table 4).

**Table 4:** Correlation of surgical ligamentum flavum thickness and area of fibrosis (%) at L4-5 and L5-S1

	L4-L5 *r*-value	*p*-value	95 % CI	L5-S1 *r*-value	*p*-value	95 % CI
Group 1	R: -0.49	R: 0.002	R: -66.27–-22.21	R: -1.00	R: 0.25	R: -191.75–131.49
	Lt: -0.30	L: <0.001*	L: -68.79–-37.32	L: -1.00	L: 0.065	L: -125.74–16.82
Group 2	Rt: -0.56	R: <0.001*	R: -57.39–-30.52	R: -0.41	R: 0.001	R: -56.30–-23.24
	L: -0.51	L: <0.001*	L: -52.02–-26.23	L: -0.53	L: 0.002	L: -72.38–-23.92
Group 3	n(1)			R: 0.95	R: 0.047	R: -111.05–-2.00
				L: 0.85	L: 0.085	L: -116.13–-17.05

*LF* ligamentum flavum, *CI* confidence onterval, %: percentage, r: correlation coefficient, * significant *p*-value, *Rt* right, *Lt* left

## Discussion

Developmental spinal stenosis is characterized by its pre-existing canal narrowing, which forms likely via a genetic origin [10, 22]. The pre-existing canal narrowing in DSS may potentiate the risk of developing stenotic symptoms, and may increase the susceptibility of spinal stenosis to be manifested with only a less degree of LF hypertrophy, as compared to the normal-sized canals. Our study, however, is the first to note that in DSS, the LF undergoes hypertrophy to a similar degree but less fibrosis as compared to non-DSS, suggesting an inverse relationship between canal size and LF fibrosis. This is in contrast to the LF of non-DSS subjects where an increase in LF fibrosis occurs. This indicates that different pathomechanisms exist for LF hypertrophy in patients with different canal diameters.

Ligamentum flavum hypertrophy is a major cause of lumbar spinal stenosis, however, the underlying cause of hypertrophy remains controversial. There are two main theories behind the appearance of hypertrophic LF. Supporting evidence of collagen synthesis and fibrotic changes are held responsible for LF hypertrophy [21, 23]. Increased expression of these biomarkers up-regulate fibroblasts for collagen formation and encourages degradation of elastic fibers [13, 24, 25]. These mechanisms are part of the degenerative process that occurs in the spine. Degenerative processes like facet hypertrophy may induce inflammatory changes that also contributes to LF hypertrophy [26]. Various stress conditions, such as hypoxia, can upregulate biomarkers and promote LF inflammation, causing degeneration in LF and its hypertrophy as well [27]. Alternatively, some suggest that LF thickening is due to disc collapse and reduced disc height, causing a secondary in-folding of the LF into the spinal canal more than an actual LF thickening [18, 28, 29]. Despite normal thickness, LF has decreased elasticity and bulges into the canal space, resulting in canal narrowing [19, 30, 31]. Thus, factors related to disc bulging [23], collapsed disc height and BMI [22, 31–34] may also be important in this pathological process. This lack of consensus in previous studies suggest that LF may have inherent differences in pathomechanisms that we are unable to fully comprehend.

Thus, our study results provide a better understanding of the contributions by a narrowed spinal canal in terms of differences in LF histology and the pathogenesis of lumbar spinal stenosis. Although it is expected for subjects in Group 1 to exhibit a milder degree of LF hypertrophy due to its narrower spinal canal and its propensity to develop symptoms, interestingly, these subjects do *not* demonstrate thinner LF. In addition to a *lack* of correlation between canal size and LF thickness, patients with narrower canals (Groups 1 and 2) had similar LF thickness as those with larger canals (Group 3). This is comparable at all levels including L4-L5 which is the most commonly involved level.

In accordance to our theories about canal size and LF thickness, a similar relationship is expected between canal size and LF histological changes. However despite comparable LF thickness among all study groups, patients with narrower canals demonstrate a significant *inverse* relationship between LF thickness and the degree of fibrosis and fibrotic area. In contrast, subjects in Group 3 demonstrate a *positive* relationship between LF thickness and fibrosis. In this group, an increasing LF thickness can be accounted by concurrent increasing fibrotic changes. Hence, it is possible that what we subclassified in this study as Group 1–3 according to canal size was compiled together for analysis in what was reported by previous studies. This can support in part to the divide in the literature regarding whether the LF undergoes fibrotic change or is only a result of disc height loss and buckling.

This effect of canal size becomes clearer when factors such as disc height and BMI are taken into consideration. All three study groups demonstrated comparable disc height and BMI, yet more subjects had anterior disc bulging in Group 3 than in the others. These findings suggest that patients without narrowing of the bony spinal canal have a greater degree of degeneration and hence the positive relationship with the fibrotic nature of the retrieved LF. Our findings therefore suggest that LF buckling is not a significant factor contributing to visualized hypertrophy on MRI. Despite less degenerative processes in Group 1's DSS subjects, there was comparable disc height and degree of LF hypertrophy with other groups. Thus, the lack of fibrotic change on histology suggests that the pathomechanism of LF hypertrophy in DSS is not as simple as has been described. Since DSS is a disorder of development, the properties of LF in DSS may not be the same as those canals we see as a result of degeneration. Similar to a maldevelopment in spinal canal size, a possible error in fibrosis development may have occurred in these subjects' LF. Therefore, what is responsible for the thickening observed requires further investigation.

The main limitation of the study was the lack of description of cellular activity and protein expression of different grades of LF fibrosis. This is necessary to understand its relationship with different canal sizes to understand the cellular pathway that causes LF hypertrophy. Nevertheless, this was the first study to have correlated LF histology with canal size and the results have helped us gain further insight into the pathogenesis of LF hypertrophy and lumbar spinal stenosis.

## Conclusions

The finding of a significant difference in the relationships of spinal canal diameter, LF hypertrophy and the development of fibrotic process within LF of DSS and non-DSS is a novel and unique discovery. The findings of this study provide the basis and the direction of future study with the focus on mapping the mechanism leading to different degrees of fibrosis according to canal size. As these changes may be a result of mal-development, determining the gene expression is also important. By exploring these details further, the pathology of lumbar spinal stenosis, specifically DSS, and LF hypertrophy can be better understood. With knowledge of the pathway leading to hypertrophy, different pathological triggers may be manipulated to alter the disease process.
